# CD1-mediated immune responses in mucosal tissues: molecular mechanisms underlying lipid antigen presentation system

**DOI:** 10.1038/s12276-023-01053-6

**Published:** 2023-09-11

**Authors:** Seohyun Kim, Sumin Cho, Ji Hyung Kim

**Affiliations:** Department of Biotechnology, College of Life Sciences and Biotechnology, Korea University, Seoul, 02841 Republic of Korea

**Keywords:** Antigen-presenting cells, Mucosal immunology

## Abstract

The cluster of differentiation 1 (CD1) molecule differs from major histocompatibility complex class I and II because it presents glycolipid/lipid antigens. Moreover, the CD1-restricted T cells that recognize these self and foreign antigens participate in both innate and adaptive immune responses. CD1s are constitutively expressed by professional and nonprofessional antigen-presenting cells in mucosal tissues, namely, the skin, lung, and intestine. This suggests that CD1-reactive T cells are involved in the immune responses of these tissues. Indeed, evidence suggests that these cells play important roles in diverse diseases, such as inflammation, autoimmune disease, and infection. Recent studies elucidating the molecular mechanisms by which CD1 presents lipid antigens suggest that defects in these mechanisms could contribute to the activities of CD1-reactive T cells. Thus, improving our understanding of these mechanisms could lead to new and effective therapeutic approaches to CD1-associated diseases. In this review, we discuss the CD1-mediated antigen presentation system and its roles in mucosal tissue immunity.

## Immunity: T cell responses to lipid antigens

Further research is needed into how certain proteins present self and foreign lipid antigens from mucosal tissues (skin, lungs, intestines) to a specific group of T cells, triggering immune responses, and how disruption to that system triggers inflammation and disease. In a review paper, Ji Hyung Kim and colleagues at Korea University in Seoul, South Korea, examined the lipid antigen presentation system mediated by CD1 glycoproteins, and highlighted how finely tuned the system is. A defect or mutation in a single factor as a result of metabolic or environmental stresses can destabilize the system, potentially driving the development of auto-immune and mucosal tissue diseases. Improved understanding of CD1-mediated immunity could provide valuable therapeutic targets for multiple health conditions. Advances in 3D cultures and organ models will boost research efforts.

## Introduction

Most studies on the role of T cells in immune responses in mucosal tissues (i.e., skin, lung, and intestines) focus on conventional T cells that are restricted by peptide-presenting major histocompatibility complex (MHC) class I and II. However, recent studies have shown that these immune responses also involve nonclassical T cells that recognize lipids presented by CD1s. CD1 is a nonclassical MHC class I-like protein, and in humans, it exists as five isoforms denoted CD1a-e. CD1a-d present self and foreign lipid/glycolipid antigens to lipid-reactive T cells^1–3^, whereas CD1e only participates in antigen processing and not presentation^4^. Lipid presentation by CD1 induces T cell responses only when all of the following processes are performed accurately: CD1 assembly, CD1 trafficking, generation of lipid antigens, extracellular and/or intracellular lipid transfer, and lipid loading and unloading on CD1s.

Lipids act as signaling molecules that shape cell proliferation, apoptosis, metabolism, and migration. Thus, lipid metabolism plays a key role in a complex signaling network that shapes both tissue homeostasis and disease^5^. Since lipids also function as antigens^6^, changes in the lipid antigen presentation system due to metabolic or inflammatory changes or external insults (e.g., infections or pollutants) could evoke pathogenic CD1-reactive T cell responses in mucosal tissues or conversely suppress the beneficial effects of these cells. Here, we will summarize 1) the molecular biology of CD1 and CD1-restricted T cells, 2) the molecules and processes that participate in the CD1-mediated antigen presentation system, and 3) how metabolic changes and pathogenic external influences could alter the lipid antigen presentation system and affect the immunopathogenesis of mucosal diseases.

## CD1 molecules, CD1-reactive T cells, and their distribution in mucosal tissue

All placental mammals bear one or more of the CD1a-e genes. The CD1a-e isoforms in humans are classified into the following three groups based on their sequence homology: group 1 incorporates CD1a-c while groups 2 and 3 contain CD1d and CD1e, respectively. Mice bear only CD1d1 and CD1d2, orthologs of human CD1d^7^. All CD1 isoforms consist of a CD1 heavy chain with α1-3 domains and a noncovalently linked beta-2 microglobulin (β2 m). The α1- α2 superdomain of CD1a-d heavy chains has an antigen-binding cleft and an A’ roof covering it. Except for CD1b, which has four pockets (A′, F′, C′, T′), the antigen-binding clefts of other CD1s have different capacities but consist of A′ and F′ pockets^8,9^.

Since CD1e does not serve as an antigen-presenting molecule^4^, the CD1-reactive T cells in humans can be divided into groups 1 and 2: both mainly express αβ T cell receptor (TCR) but also sometimes γδ TCR. The CD1 groups differ from each other in terms of expression patterns and intracellular trafficking routes, which significantly shapes the immunological functions of the T cells that recognize them^7^.

Group 2 CD1-restricted T cells, also known as NKT cells, are classified into two subsets depending on the variability of the TCR α-chain. Type I NKT cells express a variable TCR β-chain and an invariant α-chain (Vα24Jα18 in humans and Vα14Jα18 in mice); these cells are therefore also called invariant NKT (*i*NKT) cells. In contrast, type II NKT cells have a diverse TCR repertoire. NKT cells in general are activated in early immune responses and regulate other immune cells by secreting cytokines such as interferon (IFN)-γ, interleukin (IL)-4, IL-17, or IL-10^10,11^. Consequently, they are considered innate-like lymphocytes.

Group 1 CD1-restricted T cells also have diverse TCRs and secrete similar cytokines as NKT cells when confronted with the group 1 CD1–lipid complex. However, similar to conventional T cells, they appear to respond more slowly than NKT cells. For example, Felio et al. showed with transgenic (Tg) mice expressing human CD1a-c that the *i*NKT cells responded within hours to immunization with dendritic cells (DCs) pulsed with *Mycobacterium tuberculosis* (Mtb) total lipids, whereas the group 1 CD1-restricted T cells emerged after only 5 days; however, these responses were faster and more robust when the mice were challenged a second time^12^.

Lipid antigens can be from exogenous and endogenous sources, and the latter can be targets of CD1-autoreactive T cells^1,13^. Most of these T cells recognize the combined CD1-self-lipid complex but some also recognize the CD1 molecule alone. In this case, the antigen-binding cleft of CD1 is occupied with permissive lipids that are headless or have small apolar head groups that rarely protrude from the F’ portal, and the TCR lands on the A′ roof structure covering the cleft^14,15^. This mechanism means that a single CD1-autoreactive T cell clone can react to several CD1-presented lipid ligands. Both αβ- and γδ-TCRs are involved in the lipid-independent recognition of CD1^16–18^. A recent study also showed that human γδ TCRs can bind to CD1a in a lipid antigen-independent manner via a ‘sideways’ docking mode that employs the CD1 heavy chain α1 domain and β2m^19^.

In humans, all mucosal tissues contain CD1-expressing cells under homeostatic conditions. However, group 1 CD1s are expressed primarily by professional antigen-presenting cells (APCs), namely, DCs^20,21^, macrophages^22,23^, B cells^24,25^, and Langerhans cells. In contrast, CD1d is expressed not only by APCs but also by nonlymphoid cells such as endothelial cells^26^, keratinocytes^26,27^, and intestinal epithelial cells (IECs)^28–30^. The different APCs suggest that T cells that are restricted by group 1 and 2 CD1 will play quite different roles in mucosal tissues and their diseases.

Different human mucosal tissues have different cell types that express CD1 isoforms to different degrees (Table 1). First, in the skin, CD1a is highly expressed on Langerhans cells^31^ and slightly expressed on Langerin (CD207)^-^ CD14^+^ DCs^21^. CD1b-d are expressed on dermal DCs, although CD1d showed weaker expression than group 1 CD1^20^. Second, in respiratory tracts, although CD1c^+^ DCs (also referred to as BDCA-1^+^ DCs)^32^ and monocytes^33^ are the most common, Langerhans cells^34^ and CD1b^+^ alveolar macrophages^23^ are also distributed. CD1d appears not only in APCs but also in epithelial cells in mice and humans^35–37^. Third, in the intestines, group 1 CD1s are expressed relatively weakly. Langerhans cells reside in the colonic and rectal basement membrane, while CD1b^+^ and CD1c^+^ DCs are in the lamina propria^38–41^ Unlike group 1 CD1s, CD1d is expressed at high levels by the intestine. This reflects CD1d expression by IECs as well as APCs in the small intestine and colon^29,30^. Several studies have reported that CD1-expressing cells are located in other mucosal tissues, including the urogenital tract and oral cavity. For example, CD1a- and CD1d-expressing cells are found in the epithelium of the vagina^42–44^. Langerhans cells are also found in the oral cavity^45^. Together, these results imply that dysregulation of these CD1-expressing cells might alter the frequencies and function of their reactive T cells and/or mucosal tissue environment, which could lead to immunopathogenesis in these tissues.

**Table 1:** CD1-expressing cells in mucosal tissues.

Mucosal tissues	Location	CD1-expressing cell subsets	CD1 isoforms	References
Skin	Epidermis	Langerhans cells	CD1a, CD1c	^31^
	Dermis	Langerin (CD207)^-^ CD14^+^ DCs	CD1a	^21^
	Dermis	Dermal DCs	CD1b, CD1c, CD1d	^20^
	Outer epidermis	Keratinocytes	CD1d	^20,26,27^
Respiratory tracts	Lung	BDCA-1^+^ DCs	CD1c	^32^
	Lung	Langerhans cells	CD1a	^34^
	Lung	Alveolar macrophages	CD1b	^23^
	Bronchia, Bronchoalveolar lavage	CD14^+^ monocytes, Myeloid DCs	CD1c	^33^
	Lung, Bronchia	Epithelial cells	CD1d	^37^
	Lung	Alveolar macrophages, CD11b+ monocytes, CD103+ DCs, CD11b+ DCs, Epithelial cells	CD1d	^35,36^
	Bronchoalveolar lavage	Macrophages	CD1d	^35^
Intestines	Lamina propria of colon	CD19^-^ myeloid DCs	CD1c	^38^
	Basement membrane of sigmoid colon and rectum	Langerhans cells	CD1a	^39^
	Lamina propria of sigmoid colon and rectum	DCs	CD1b, CD1c	^39,40^
	Duodenum	CD103^+^ CD11c^+^ DCs	CD1c	^41^
	Small intestine, Colon	Epithelial cells	CD1d	^29,30^
Others	Vagina	Epithelial cells	CD1d	^44^
	Vagina	Vaginal epithelial DCs, Vaginal Langerhans cells	CD1a	^42,43^
	Oral epithelium	Langerhans cells	CD1a	^45^

## Assembly and trafficking of the CD1–lipid complex

**Fig. 1:**
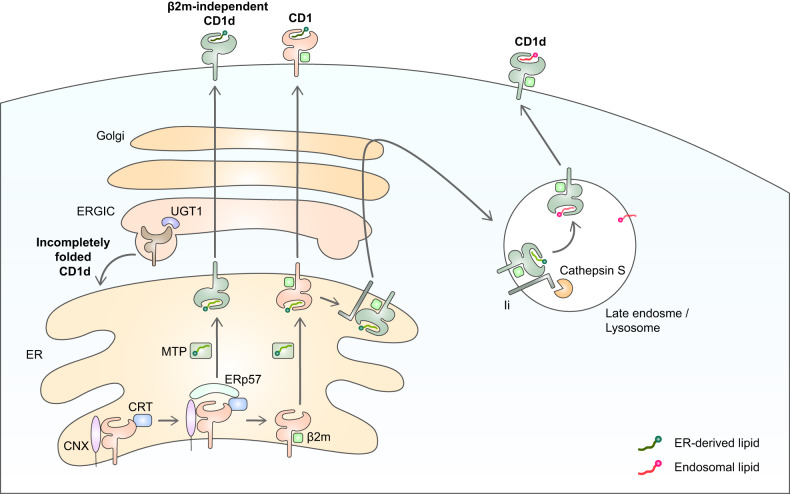
CD1 assembly and lipid loading on native CD1. CD1 is assembled in the ER. To induce proper CD1 heavy chain folding, CRT and CNX bind simultaneously to the heavy chain, after which ERp57 is recruited. For CD1d, the CRT/CNX quality control pathway is regulated by UGT1, which is located in the ER-Golgi intermediate compartment (ERGIC). UGT1 reglucosylates the incompletely folded CD1d heavy chain so that the ER chaperones bind to CD1d again. Once CD1 is fully folded, β2 m binds noncovalently, and the CD1-β2 m heterodimer loads endogenous placer lipids derived from the ER. Lipid transfer is mediated by MTP. The loaded complex passes through the Golgi and travels to the cell surface, where it presents itself to the extracellular environment. However, some CD1d molecules do not exactly adhere to this standard process. Thus, a small number of CD1d molecules do not bind to β2 m but nonetheless receive lipids and egress the ER. Moreover, some CD1d molecules bind to the invariant chain in the ER, and instead of visiting the cell surface first, they migrate directly to the LE compartment. There, the CD1d molecules exchange their ER-derived lipids with endosomal lipids. A protease named cathepsin S then processes the invariant chain, allowing for the transfer of CD1d to the cell surface. This event occurs independently of CD1d recycling and increases the exposure of CD1d to endosomal lipids.

CD1 heavy chain molecules are assembled in the endoplasmic reticulum (ER). As with MHC class I and II molecules, ER chaperones control the beginning of CD1 assembly. Indeed, CD1 assembly is controlled by the same chaperones that guide MHC class I assembly, namely, the lectin chaperones calreticulin (CRT) and calnexin (CNX) and the thio-disulfide oxidoreductase ERp57. However, there are several differences between MHC class I and CD1 assembly. Unlike MHC class I, where CRT and CNX are sequentially attached^46,47^, two chaperones bind to the CD1 molecule simultaneously to form a ternary complex, followed by the recruitment of ERp57 to create a disulfide bond in the heavy chain^48^. Disulfide bond formation was impaired when the association was inhibited by treatment with glucosidase inhibitors since the lectin chaperone is associated with the heavy chain through an *N*-linked glycan moiety^49^. Once the CD1-heavy chain is fully folded, it then generally binds to β2 m. At this point, endogenous lipids (e.g., phosphatidylinositol^50^) in the ER are loaded into its binding cleft, and the complex leaves the ER and travels to the cell surface through the Golgi apparatus. It should be noted, however, that while group 1 CD1s can only exit the ER when the β2m-CD1 heterodimer has formed^9,51,52^, this is not necessarily true for CD1d; CD1d molecules that bear immature glycosylation patterns and lack β2 m are common on the plasma membrane^53,54^. Notably, this does not seem to affect the ability of the cells to activate NKT cells^55–57^. CRT seems to play a role in the ER retention of CD1d since CRT-deficient cells bear more β2m-free CD1d heavy chains on their surface. However, most of these CD1d heavy chains are rapidly internalized and degraded in lysosomes, which suggests that β2 m prevents the lysosomal degradation of the CD1d heavy chain^58^. If poorly loaded MHC class I molecules are released prematurely, a rescue mechanism occurs; here, UDP-glucose glycoprotein glucosyltransferase-1 (UGT1) in the ER and ER-Golgi intermediate compartment selectively reglucosylated an *N*-linked glycan on the heavy chain, which returns it to the ER^59,60^. UGT1 also participates in CD1 folding since UGT1-deficient cells show premature formation of CD1d-lipid complexes in the ER that are associated with a shortened CD1d-complex half-life on the cell surface and altered antigenicity^61^ (Fig. 1). The role of UGT1 in group 1 CD1 folding has not been studied.

It should be noted here that the endogenous lipids that are loaded in the CD1 molecules in the ER are later replaced with other endogenous and exogenous lipid antigens, either at the cell surface^62,63^ or in endosomal compartments^64–66^. Indeed, because CD1 molecules undergo lipid exchange and recycling back to the plasma membrane, a single CD1 molecule can present multiple lipid antigens over its lifetime.

**Fig. 2:**
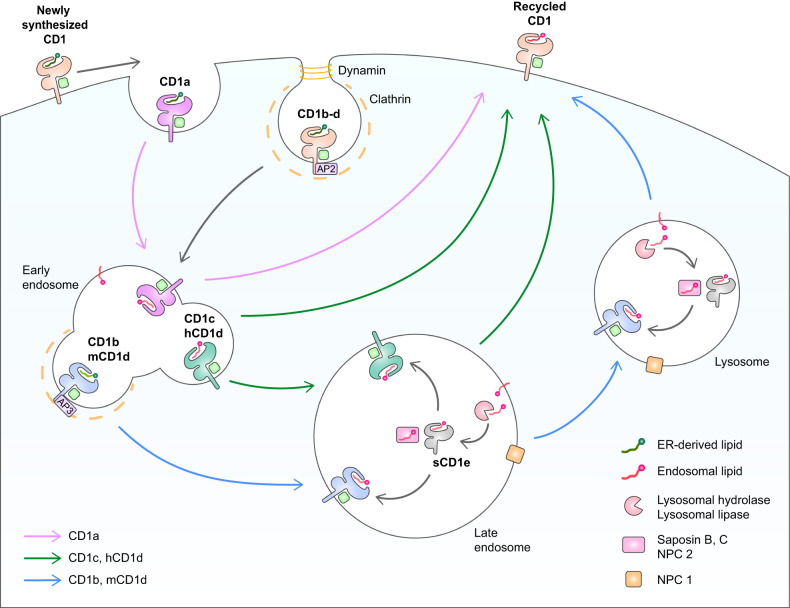
CD1 recycling and lipid exchange. The CD1a-d isoforms are all expressed on the cell membrane. From there, all but a few CD1a molecules undergo subsequent intracellular trafficking to exchange their ER-derived lipids with processed lipids in the endosomal/lysosomal compartments before returning to the cell surface. The few CD1a exceptions exchange their placer lipids with exogenous lipids. The remaining CD1a molecules follow a simple trafficking pathway into early endosomes. This reflects the fact that the cytoplasmic tail of CD1a lacks the tyrosine-based sorting motif that would send it to the LE/lysosome. In contrast, CD1b-d bear the AP2-binding motif in their cytoplasmic tails. Thus, they accumulate in clathrin-coated pits or vesicles and then move to the early endosome by clathrin- and dynamin-dependent mechanisms. Thereafter, CD1b and mouse CD1d (mCD1d), which bear an AP3-binding motif in their cytoplasmic tails, are sorted to the LE and lysosome. In contrast, human CD1d (hCD1d) and most CD1c molecules, which lack AP3 in their cytoplasmic tails, undergo lipid exchange in the early endosome. However, a few CD1c molecules can also migrate to the LE via an AP3-independent mechanism. Since the lipids with which the placer lipids are exchanged in the LE compartment are strongly shaped by the enzymes in this compartment, these enzymes play an important role in the antigen repertoire that is presented by recycled CD1. The enzymes include lysosomal hydrolases that catabolize carbohydrate moieties of endosomal lipids. These hydrolases include α-mannosidase, acid ceramidase, α-galactosidase A, α-glucosidase, and ceramide synthase-2. Lysosomal lipases such as PLA2, which digest lipid moieties, are also important. Lipid-transfer proteins also play an important role in lipid exchange in the LE/lysosome by helping load lipids into the CD1-binding groove. These lipid-transfer proteins include saposin B, saposin C, NPC1, and NPC 2. Another important molecule is soluble CD1e (sCD1e). These molecules do not present lipid antigens to T cells such as CD1a-d; rather, they assist in the processing of lipids and the CD1-loading of lipids in endosomes.

At the cell surface, the lipid inside the CD1 molecule can be exchanged directly with soluble exogenous lipids. This appears to be a key mechanism by which CD1a obtains its antigens^62^. CD1c showed a similar lipid exchange pattern to CD1a^63^. However, since CD1b-d molecules are internalized with the help of adaptor protein complex (AP)2 bound to the sorting motif on the cytoplasmic tail, it is likely that lipid exchange during intracellular trafficking is the predominant mechanism by which these CD1s acquire their antigens. CD1 isoforms internalized by clathrin- and dynamin-dependent mechanisms are first located in the early endosome^66–68^, and what happens next depends on whether AP3 binds to them. Because they lack tail motifs that bind to AP3, most CD1c and human CD1d molecules remain in the early endosome. In contrast, CD1b and mouse CD1d molecules bear the AP3-binding sorting motif and are therefore actively redirected into the late endosome (LE)/lysosome^65,69–72^. The remaining CD1c molecules are also transferred to lysosomes, but in an AP3-independent manner. However, more than half are located at the internal membrane of the lysosome, whereas CD1b is mostly detected at the limiting membrane. This suggests that the few CD1c molecules that are directed to lysosomes may differ from CD1b in terms of the lipids that are loaded^73^. It should be noted that CD1a can also be internalized, after which it is quickly distributed to early endosomes and then to recycling endosomes^74^. The mechanism by which CD1a molecules internalize remains to be determined, but their recycling to the cell surface may involve Rab22a and ADP ribosylation factor-6^75^. Interestingly, some CD1d molecules enter the LE and lysosome without being expressed on the surface first. This is directly mediated by the association between CD1d and invariant chain (Ii) glycoprotein. Ii is better known for its key role in exporting MHC class II molecules from the ER to the LE through the Golgi apparatus. Ii is then degraded by the lysosomal cysteine proteases cathepsin S and L^76,77^. Notably, these proteases are involved not only in MHC class II maturation but also in the development and activation of NKT cells. Cathepsin S may play a key role in the endosomal trafficking of CD1d since it is disrupted in cathepsin S-deficient DCs^78^, and this effect is not observed for cathepsin L^79^. Thus, CD1d can gain its antigens by two trafficking mechanisms (Fig. 2). These mechanisms are nonredundant and are thought to stabilize endosomal lipid loading events^80,81^.

## Lipid antigens that trigger CD1-mediated immune responses

The lipid antigens that replace the endogenous spacer lipid in the CD1-binding groove after the molecules leave the ER include exogenous antigens from infectious organisms or endosymbionts, allergens, and other endogenous lipids.

The best-studied infection-derived antigens are membrane lipids from mycobacteria; these include dideoxymycobactin^82^, glucose monomycolate^83^, mannosyl-1B-phosphomycoketide^84^, and phosphatidylinositol mannoside-4^85^ and are recognized by CD1a, CD1b, CD1c, and CD1d, respectively. The core structure of these lipids has not been found in mammals, which suggests that CD1 may have partially evolved to stimulate T cell responses against mycobacteria^12,86^. Lipid antigens for CD1 have been found in other pathogenic bacteria, including *Borrelia burgdoferi*^87–89^, *Streptococcus pneumoniae*^90^, and *Staphylococcus aureus*^91^.

The lipid antigens from *S. aureus* include phosphatidylglycerol (PG) and lysylPG; interestingly, it was recently shown that these molecules can activate CD1a-reactive T helper (Th)-2 type immune responses. Since skin colonization with *S. aureus* is associated with atopic dermatitis (AD) and AD patients have higher CD4^+^ CD1a-(lysyl)PG tetramer^+^ T cell frequencies in their peripheral blood than normal controls, it is possible that PG-induced CD1-reactive T cells contribute to AD pathology^92^. Other endosymbionts also bear lipid antigens that are the focus of CD1-reactive T cell responses. For example, glycosphingolipids (GSLs) from intestinal opportunistic microbes can stimulate *i*NKT cells in a CD1d-dependent manner^93–95^. However, microbial GSLs can also inhibit the development of gut NKT cells^95,96^. Thus, gut bacteria and NKT cells may interact in complex ways to influence each other.

Multiple plant-derived lipid allergens have been identified. They include urushiol in poison ivy, which causes allergic contact dermatitis (ACD). This is partially mediated by the presentation of pentadecylcatechol (C15:2) in urushiol by CD1a since group 1 CD1-deficient wild-type mice demonstrate milder urushiol-induced ACD than CD1a-transgenic mice^97^. Similarly, farnesol, an ingredient in cosmetics that can cause severe ACD, can bind to CD1a^98^. Phospholipids in cypress pollen may also be the cause of allergy to this pollen; the CD1a- and CD1d-restricted T cells in the blood of cypress pollen-sensitive subjects have more cytokine production and proliferation in response to pollen-derived phospholipids than equivalent cells from control subjects^99^.

The endogenous lipids that arouse CD1-restricted T cell responses include phospholipids^100^, GSLs^101,102^, and cholesteryl ester^88^. In healthy conditions, most of these self-lipids are in cell membranes or organelles. Sebaceous-gland lipids such as squalene and wax esters in the extracellular space can also bind to CD1a. These lipids lack a polar head group that protrudes outside of CD1a and can be recognized by CD1a-autoreactive TCRs^103^. Other pathogenic CD1-binding lipids may arise during specific conditions that induce the overexpression of self-lipid antigens; these conditions include ER/mitochondrial stress^104–106^, bacterial infection^107^, and exposure to specific allergens^108,109^.

## Proteins that mediate the generation of endo/exogenous lipid antigens presented by CD1

To be able to bind to CD1, lipid antigens must generally first be synthesized or taken up by the cell through cell-surface receptors or membrane internalization. The role of the latter in shaping lipid antigen uptake has been extensively reviewed by Sugita et al.^110^. Therefore, we will not discuss this further. In many cases, the antigens then have to be processed into forms that can bind to CD1. Therefore, this section focuses on the enzymes and nonenzymatic proteins that drive lipid antigen synthesis and modification.

### Endogenous ligands

Isoglobotrihexosylceramide (iGb3) is presented by CD1d and recognized by *i*NKT cells. β-hexosaminidase generates the lysosomal GSL iGb3 from iGb4. Mice lacking the β-subunit of β-hexosaminidase were unable to generate *i*NKT cells^102^. α-galactosidase A is a rate-limiting enzyme that catalyzes the degradation of iGb3 to lactosylceramide. Mice that lack α-galactosidase A demonstrated *i*NKT cell overactivation due to the accumulation of iGb3^111^. Due to these studies, iGb3 was considered to be one of the endogenous ligands needed for *i*NKT cell development. However, there is a report showing that iGb3 synthase-deficient mice did not affect the *i*NKT TCR repertoire and that α-galactosidase A deficiency in mice led to a reduction in *i*NKT cells due to dysfunction of globoside storage, implying the existence of another ligand that is necessary for the development of *i*NKT cells^112^.

α-galactosylceramide (α-GalCer) is the prototypical *i*NKT cell activating ligand^113^. It had been thought that mammals only bared β-linked glycosylceramides until Kain et al. showed that mammalian immune cells constitutively produce very tiny amounts of α-glycosylcermides. They also showed that the production of α-glycosylcermides by these cells is tightly controlled by catabolic enzymes, including acid ceramidase and α-glycosidase. Indeed, blocking these enzymes induced the accumulation of α-glycosylceramides in vitro^114^. However, the synthesis pathway and regulation of α-glycosylcermides remain to be determined.

Recently, Saroha et al. showed that very-long acyl-chain sphingolipids play important roles in the development and maturation of *i*NKT cells in the thymus and that these roles could be blocked by ceramide synthase 2 deficiency in mice^115^.

Several studies have also found that endogenous multi-acylated glycolipids and phospholipids must be digested by lysosomal lipases before they activate *i*NKT cells. An example of these lipases is lysosomal phospholipase A2 (PLA2), which is localized to the LE/lysosome and acts as both a phospholipase and transacylase^116^. Lysosomal PLA2-deficient mice had diminished *i*NKT cell numbers and altered presentation of endogenous or exogenous antigens that require endocytic processing^117^.

### Exogenous ligands

Hexamannosylated phosphatidyl-myo-inositol (PIM6) is from mycobacteria and must be hydrolyzed by lysosomal α-mannosidase before it can be recognized by CD1b-restricted T cells. Notably, CD1e acts as an essential accessory protein in this processing event by binding glycolipids and assisting in digestion^4^. A follow-up study showed that CD1e selectively aided in the processing of diacylated PIM6^118^.

Another study showed that lysosomal PLA2 and pancreatic lipase-related protein-2 play essential roles in the presentation of antigenic PIMs by CD1b in vivo. This role is mediated by their digestion of PIM to diacylated PIM^119^.

## Lipid transfer proteins that shape CD1-mediated antigen presentation

The ER not only synthesizes the vast majority of cellular lipids but is also where nascent CD1 molecules incorporate their spacer lipids. ER proteins that participate in the exchange and/or transfer of lipids can shape CD1-mediated antigen presentation. One of these is microsomal triglyceride transfer protein (MTP), which is a lipid transfer protein that resides in the ER and is essential for the lipidation of apolipoprotein B^120^. Several studies have suggested that MTP plays a key role in loading lipid antigen onto nascent CD1d molecules in the ER of CD1d-expressing cells^121–123^. When the MTP-encoding gene was deleted in hepatocytes or IECs, cell-surface expression of CD1d and activation of NKT cells were reduced^121^. A follow-up study then showed that purified MTP directly transferred phospholipids onto recombinant CD1d in vitro^122^. MTP may also play another role in CD1d antigen presentation; Sagiv et al. found that MTP deficiency impaired the trafficking of CD1d from the lysosome to the plasma membrane, although the mechanism remains to be characterized^123^. MTP also regulates group 1 CD1-mediated antigen presentation; blocking MTP function with inhibitor or RNA interference-mediated silencing decreased exogenous mycobacterial antigen presentation by CD1b and CD1c, which hampered the activation of group 1 CD1-restricted T cell clones that recognize the mycobacterial antigen^124^.

Lipid transfer proteins in the endocytic pathway, especially the lysosome, also assist lipid antigen presentation by CD1. Saposins A-D are generated from prosaposin by endosomal proteolytic cleavage. These molecules serve as cofactors that promote GSL hydrolysis in the lysosome^125^. The importance of saposins in CD1 biology was suggested by the finding that prosaposin-deficient mice have defective CD1d-mediated antigen presentation and thymic Vα14^+^ NKT cell development^126^. Moreover, the lysosomal binding of α-GalCer to CD1 in prosaposin-deficient CD1-expressing fibroblasts only occurred when they were transduced to express prosaposin. However, the absence of prosaposin did not alter intracellular CD1 trafficking^127^. The role of saposins in CD1 antigen presentation is itself regulated by other molecules, including cathepsin D. This is an aspartyl protease in APC lysosomes that cleaves prosaposin into saposins. When DCs were treated with a cathepsin D inhibitor, prosaposin cleavage decreased along with the ability of the DCs to induce *i*NKT cell expansion^128^.

Specifically, saposin B enhances CD1d-mediated endogenous/exogenous lipid antigen presentation. In vitro assays showed that saposin B directly mediates lipid binding to CD1d and that lipid-laden saposin B increases the off-rate of lipids bound to CD1d, which suggests that it helps unload lipids from CD1d^129,130^. Moreover, saposin C is needed for CD1b presentation of mycobacterial lipids because it extracts these antigens from the intralysosomal membrane^131^ (Fig. 2). The roles of saposin A and saposin D in CD1-mediated antigen presentation have not yet been delineated.

CD1e not only aids protein processing but is also a lipid transfer protein that facilitates the formation of CD1–lipid complexes in the LE/lysosome. Specifically, it promotes CD1–lipid loading and unloading. However, CD1e does not affect the presentation of all lipids, which suggests that this effect depends on the binding affinity of lipid antigens to CD1e^132^.

Other lipid transfer proteins of note are Niemann-Pick type C (NPC)1 and 2. These are LE/lysosomal glycoproteins that participate in the trafficking of GSLs and cholesterol^133,134^. NPC1-deficient and NPC2-deficient mice lack Vα14^+^ NKT cells; moreover, CD1d-expressing APCs that are deficient in NPC1 and NPC2 demonstrated impaired lipid antigen presentation. NPC1 deficiency appears to interfere with the transfer of lipid antigens from the LE to the lysosome^135^. One study suggested that this mode of action is not the case for NPC2 deficiency; rather, it seemed to decrease the efficiency with which lipids are loaded onto and unloaded from CD1d^136^. However, another study found that NPC2 deficiency may act more indirectly by inducing the lysosomal accumulation of lipids, which alters the repertoire of lipid antigens that are presented to NKT cells^137^. In contrast, a human study on NPC disease, which is caused by NPC1 and NPC2 defects, did not find that these defects decreased CD1d-mediated antigen presentation^138^.

## Regulation of CD1-mediated lipid antigen presentation by cellular metabolism

CD1 transcription levels in APCs directly shape CD1-reactive T cell activity and are regulated by various transcriptional regulators. Multiple studies have suggested that cellular metabolism regulators play a particularly key role in shaping CD1 expression. One is peroxisome proliferator-activated receptor (PPAR)γ, which, similar to other PPARs, is a member of the nuclear receptor superfamily. PPARs are activated by dietary fatty acids and eicosanoids and play crucial regulatory roles in cellular metabolism by inducing the expression of enzymes involved in lipid metabolic pathways^139^. PPARγ plays an important role in CD1 expression by DCs. For example, when human monocytes were induced to differentiate in vitro into immature and mature DCs, PPARγ was immediately induced. PPARγ then enhances lipid uptake, upregulates CD1d expression, and downregulates the expression of group 1 CD1s^140^. The differential expression of CD1d and CD1a is mediated by PPARγ-induced expression of retinol and retinal-metabolizing enzymes that generate all-trans retinoic acid; this activates the transcription factor retinoic-acid receptor-α (RARα), which in turn rapidly upregulates CD1d and downregulates CD1a expression^141^.

Notably, when monocytes are differentiated into DCs in vitro, a mixed population of CD1a^-^ DCs and CD1a^+^ DCs arises. CD1a^-^ and CD1a^+^ DCs differ in the cytokines and chemokines they produce and therefore their T cell polarizing potential. The transition from CD1a^-^ to CD1a^+^ DCs is associated with the reduced uptake of lipids and downregulated expression of PPARγ and lipids. Interestingly, serum lipoproteins promote the generation of the CD1a^-^ DCs and block the production of CD1a^+^ DCs. This suggests that the lipid environment could modulate DC functions, including antigen uptake and presentation^142^. Indeed, oxidized-low density lipoprotein and human serum have been shown to contain PPARγ activators that upregulate PPARγ expression and retinoid signaling in DCs; therefore, PPARγ also regulates their expression of CD1^140–142^. Moreover, lysophosphatidic acid and cardiolipin in human serum are PPARγ ligands that downregulate the expression of group 1 CD1s in monocyte-derived DCs^143^.

PPARγ also regulates CD1 expression in other APCs, including B cells. However, unlike monocyte-derived DCs, this does not always involve RARα signaling. Thus, while human B cells constitutively express CD1c and CD1d, the expression of both are downregulated when the cells are activated by CD40L alone. This is associated with lower RARα transcriptional activity. In contrast, when the B cell receptor is stimulated with or without CD40L, CD1c expression is enhanced, whereas CD1d expression drops. This does not involve changes in RARα activity^144^.

CD1 expression is also regulated by 5’-AMP-activated protein kinase (AMPK), which is a master regulator of metabolism. When cellular ATP levels decrease, AMPK is activated and suppresses anabolic activities, including lipid and protein synthesis, and enhances catabolic activities, including lipid oxidation and glucose metabolism to restore energy homeostasis^145^. Webb et al. showed that this can also affect CD1d expression as follows: pretreatment of APCs with AMPK activators prevented lymphocytic choriomeningitis virus infection-induced upregulation of CD1d and reduced their ability to activate NKT cells. This finding also suggests that stressors such as viral infections could alter APC metabolism, thereby altering CD1d-mediated immune responses^146^.

Another such stressor is ER stress, which is characterized by the accumulation of abnormal misfolded or unfolded proteins. When ER stress arises, the ER seeks to restore homeostasis by evoking the unfolded-protein response^147^. A key sensor of ER stress is PKR-like ER kinase (PERK), which is localized in the ER membrane and reduces RNA translation when it encounters unfolded proteins. When human and mouse-derived APCs undergo ER stress, they increase endogenous lipid antigen presentation by CD1d, which activates *i*NKT cells. This activation depends on PERK^104,106^. The underlying mechanism is not known, but one study suggested that ER stress affects actin cytoskeletal reorganization, which contributes to CD1d expression on the cell surface^104,106^. Alternatively, it could reflect de novo expression of endogenous lipids; ER stress can alter the transcription of lipid metabolic proteins^147^. Indeed, ER-stressed APCs demonstrated transcriptional downregulation of GSL catabolic enzymes and therefore the accumulation of endogenous GSL^104,106^. However, not accumulated GSLs, neutral lipids were the lipids that activate *i*NKT cells in this setting^104^. Whether the generation of neutral lipid antigens during ER stress results from de novo synthesis or altered lipid loading processes remains undetermined^104^. These observations are interesting because ER stress in various cells plays an important role in the development of diseases such as inflammation and cancer^148^; thus, it is possible that this role is in part mediated by ER stress in APCs, which alters their CD1d-mediated antigen presentation and thereby shapes *i*NKT cell functions. However, determining whether ER-stressed APCs also induce altered group 1 CD1-mediated immune responses requires further investigation.

Mitochondrial stress is another stressor that can change APC metabolism and thereby shape CD1-mediated immune responses. For example, mitochondria synthesize phosphatidylglycerol in low amounts under normal conditions. However, during mitochondrial stress, phosphatidylglycerol levels rise and it may escape to membranes that bear CD1b; this lipid is loaded by CD1b, which then acts as a self-antigen that induces CD1b-restricted T cells^105^.

Collectively, these results suggest that CD1-mediated antigen presentation can be regulated by cellular metabolism. Notably, mucosal tissue diseases such as inflammatory diseases and infections involve metabolic alterations; it is possible that these adaptations could lead to lipid antigen presentation changes based on alteration of the generation of lipid antigens, CD1 expression, and/or CD1 trafficking. This suggests that the metabolic pathways that shape lipid antigen presentation could be novel therapeutic targets for mucosal tissue immune diseases.

## Role of altered CD1-mediated lipid antigen presentation in mucosal tissue immune diseases

CD1-mediated immune responses regulate mucosal tissue immunity, which can be regulated by inflammation and infection. Improving our understanding of the mechanisms that control lipid antigen presentation in the context of mucosal tissue diseases may help identify novel therapeutic targets. These possibilities are explored below.

### Skin diseases

PLA2s are derived not only from endogenous origins but also from foreign origins, and wasp venom contains PLAs that can promote the activation of CD1a-restricted T cells, which are abundant in the skin and promote allergic inflammation of the skin after bee/wasp stings. Bourgeois et al. found that PLA2s in bee and wasp venom cleave nonantigenic phospholipids in the venom and skin into lysophospholipids and antigenic fatty acids, which are then presented by CD1a and activate CD1a-restricted T cells^108^. Indeed, their follow-up study showed that compared to nonallergic individuals, venom-allergic individuals had higher circulating frequencies of venom-specific CD1a-restricted T cells that produced IFN-γ, GM-CSF, and IL-13^149^.

Foreign PLA2 may also participate in AD, which is a chronic inflammatory disease that causes the skin to become itchy, red, and swollen. It is caused by genetic, immunological, and environmental factors^150^. A classical environmental factor is house-dust mite extract (HDM). Jarrett et al. showed that HDM contains PLA2, which is active in HDM-exposed human skin. This appears to generate antigenic lipids that are recognized by CD1a-restricted T cells; these cells are enriched in the blood and skin of AD patients, produce more Th2 cytokines, and infiltrate the skin after HDM challenge^109^. Notably, endogenous PLA2 may also participate in CD1a-restricted T cell responses to HDM. Some type-2 innate lymphoid cells (ILC2s), which reside in barrier sites and are involved in Th2-type inflammatory responses^151^, express CD1a and can activate CD1a-restricted T cells. ILC2s express PLA2G4A, a cytoplasmic PLA2 that has been shown to help generate neolipid antigens during HDM challenge^152^. This ILC2-based mechanism also participates in the neolipid antigen presentation that occur during *S. aureus* infection^152^.

Psoriasis is an autoimmune skin disease that is associated with the Th1 and Th17 responses. Significantly, psoriasis pathogenesis may also depend on CD1a; when imiquimod-treated CD1a-transgenic mice, which are a model of psoriasiform inflammation, were treated with anti-CD1a, their amount of skin inflammation dropped significantly^97^. The relationship between PLA2G4D, a cytoplasmic PLA2, and psoriasis was shown by Cheung et al. They found that mast cell-derived PLA2G4D was increased in psoriatic plaques, although unexpectedly, its activity was extracellular. It was then found that this reflected the transfer of PLA2G4D to CD1a-expressing cells via exosomes. This lead to the generation of neolipid antigens that are then presented on CD1a, which activate CD1a-restricted T cells and cause them to produce IFN-γ, IL-17, and IL-22^153^.

A lipase that has PLA2 activities^154^, namely, acyloxyacyl hydrolase (AOAH), may also participate in psoriasis. This lipase usually plays a pivotal role in the detoxification of lipopolysaccharides. Singh et al. showed that psoriatic lesions, but not healthy skin, express the AOAH protein. A closer analysis revealed that this AOAH comes from CD1a-expressing cells as well as phagocytic cells such as neutrophils and macrophages. Moreover, AOAH generates neolipid antigens that are presented by a CD1a-expressing APCs, which can activate autoreactive CD1a-restricted Th17 cells from the blood. Moreover, analysis of these cells showed that they expressed higher levels of IL-22 when they came from psoriasis patients. Thus, the PLA2 activity of AOAH may also contribute to psoriasis pathogenesis^155^.

The notion that lipid metabolism dysregulation alters CD1-mediated immune responses and thereby promotes psoriasis is further supported by the finding that dyslipidemia is a risk factor for psoriasis patients^156^ and hyperlipidemic Tg mice that produce a CD1b-autoreactive T cell clone spontaneously developed psoriasiform dermatitis^157^. The plaques in these mice preferentially accumulated phospholipids and cholesterol that could directly activate the autoreactive T cell clone. Additionally, hyperlipidemic serum enhanced IL-6 secretion by CD1b^+^ dermal DCs and thereby increased IL-17A production by the T cell clone. Indeed, psoriasis patients bear more CD1b-expressing cells and CD1b-autoreactive T cells in their blood than healthy individuals^157^.

KCs are found in psoriatic plaques of patients with CD1d overexpression. It was confirmed that NKT cells secrete large amounts of cytokines such as IFN-γ and IL-13 when cocultured with CD1d-expressing KCs^26,158^, despite the very low frequency of NKT cell infiltration into the psoriatic lesion^159^. Since these cytokines again promote the activation and differentiation of KCs^160^, the role of CD1d and NKT cells in psoriasis development cannot be overlooked.

ACD is a delayed-type hypersensitivity response that involves CD1-reactive T cells as well as conventional T cells. A classical trigger is dinitrochlorobenzene (DNCB). When CD1d-expressing APCs were treated with DNCB in vitro, they can activate a CD1d-restricted T cell clone. This activation event depends on not only the new synthesis of CD1d molecules but also endogenous lipids in the APCs. Notably, other contact sensitizers (resorcinol, isoeugenol, and cinnamaldehyde) can also trigger the activation of the CD1d-restricted T-cell clone. This suggests that DNCB and the other sensitizers act via the same mechanism. It was speculated that this similarity could be related to the induction of self-antigens. Alternatively, the mechanism could involve the binding of sensitizer to nascent CD1d molecules, which would alter the lipid repertoire that can bind to the binding cleft^161^.

Similarly, the lipophilic environmental pollutant benzo[a]pyrene, which promotes multiple inflammatory diseases, including allergic inflammation, autoimmune diseases, and cancer, may impair lipid antigen presentation by altering the expression of genes in the endocytic and lipid metabolic pathways, resulting in decreased expression of CD1a and CD1d on human DCs^162^.

### Respiratory diseases

The fact that the lung expresses CD1s suggests that these molecules may also participate in respiratory diseases. Indeed, *i*NKT cells are relatively more frequent in the lung than in the peripheral blood and play key roles in airway hyperreactivity (AHR), which is a hallmark of allergic asthma^163,164^. Moreover, as shown by β2m-knockout mice, noninvariant NKT cells that recognize β2m-independent CD1d also participate in the development of AHR in ovalbumin-induced asthma mouse models; treatment of these mice with anti-CD1d decreased AHR^57^. Bansal et al. showed that cockroach extract exposure, which induces NKT cells to drive allergic asthma, elevates secretory PLA2 production in the airways of the model mice. This enzyme induces the production of lysophosphatidylcholine, which amplifies inflammation. It was also ameliorated by blockade of CD1d^165^.

Studies in human patients with allergic asthma confirm the importance of CD1 in this disease. Thus, transcriptomics showed that Th2-type inflammation in these patients is associated with group 1 CD1-expressing DCs in the sputum^166^. Moreover, the bronchial mucosa and sputum of asthma patients bear significantly higher frequencies of CD1a^+^ DCs and CD1c^+^ DCs than equivalent samples from healthy individuals^167,168^. However, the role of these cells in asthma remains to be determined.

Smoking is a common cause of chronic obstructive pulmonary disease (COPD)^169^. Several lines of evidence suggest that the underlying mechanism could involve promoting CD1-reactive T cell responses. A cigarette smoke-exposed COPD mouse model demonstrated that model mice had elevated CD1d expression by alveolar macrophages and DCs. In humans, activated NKT cells are more frequent in the circulation of COPD patients than in healthy controls^170^. Additionally, culture with cigarette smoke extract directly activates both human airway epithelial cells and DCs, which induces their ability to stimulate *i*NKT cells to produce IL-17 and IFN-γ^170^. In humans, alveolar macrophages and monocyte-derived macrophages from COPD patients and smokers display increased CD1b expression^23^. Importantly, human bronchial epithelial cells that are exposed to cigarette smoke extract bear more oxidized lipids than control cells^23^. Thus, smoke exposure may alter the lipid repertoire that is expressed by CD1b-expressing APCs, which arouses CD1b-restricted T cell responses that participate in COPD pathogenesis.

### Gastrointestinal diseases

CD1 expression is associated with genetic gastrointestinal diseases and inflammatory bowel diseases. Abetalipoproteinemia (ABL) is a rare inherited disorder that is caused by a genetic deficiency in MTP. It affects the gastrointestinal tract and causes symptoms such as diarrhea and vomiting. Consequently, in ABL, group 1 CD1 levels are reduced because of increased proteasomal degradation, which leads to low cell-surface expression. Moreover, although the cell-surface expression of CD1d is not altered, these molecules cannot load antigens^171^. Thus, the MTP is a distinct regulator of CD1-mediated immune responses.

Notably, MTP can also play pathogenic roles in the gut; MTP depletion in the intestine suppresses oxazolone-induced colitis in mice, where it blocks CD1d-mediated antigen presentation^121^. However, if MTP is specifically depleted in IECs, mice develop severe NKT cell-mediated colitis. This is due to engagement of CD1d on the IEC surface, which induces a signaling cascade that activates the STAT3 pathway and thereby causes these cells to produce IL-10, which plays a critical role in downregulating intestinal inflammation^172–174^.

Regarding group 1 CD1s, it is possible that CD1a is a biomarker of ulcerative colitis; ulcerative colitis patients have higher frequencies of CD1a^+^ macrophages and monocytes in their blood than healthy individuals^22^. However, further investigation of the roles of CD1b and CD1c expression and group 1 CD1-reactive T cells in colitis is needed.

### Bacterial and viral infections

Since bacteria and viruses invade the body through barrier sites and these sites express more CD1 than the blood, it is likely that CD1-mediated immune responses play pivotal roles in protecting the host from these pathogens. Indeed, such infections strongly induce these immune responses via various mechanisms.

First, bacterial infections can induce the synthesis of CD1 molecules. For example, Mtb infection of CD1^-^ myeloid precursors triggers the expression of group 1 CD1 proteins on their surface and the surface of bystander cells. This is due to polar lipids from Mtb, which activate Toll-like receptor (TLR)2 signaling and upregulates the transcription of group 1 CD1s^175^. Similarly, in vitro analyses of the blood monocytes of *Mycobacterium leprae*-infected patients show that for differentiation into CD1b^+^, DC expression of CD1b is induced by TLR2/1-mediated elevation of GM-CSF, which promotes T cell activation^176^. Interestingly, *M. leprae* may evade CD1b-restricted T cell responses by inducing the macrophage accumulation of host-derived oxidized phospholipids that impair their differentiation into CD1b^+^ DCs; this inhibitory effect could be overcome by adding normal HDL, which is a scavenger of oxidized lipids, to the culture. However, HDL from patients with leprosy did not have this effect, although the underlying mechanism remains to be determined^177^. Notably, Mtb infection of myeloid precursors does not upregulate the expression of their CD1d transcripts or proteins^175^. However, CD1d expression can be upregulated when macrophages are cultured with Mtb products together with IFN-γ^178^.

The second mechanism by which microorganisms induce CD1-mediated T cell responses is by the presence or production of antigens that bind to CD1 during infection. The CD1-binding antigens include not only the pathogen-derived exogenous lipids but also endogenous GSLs that are synthesized de novo in APCs after infection. For example, when DCs are infected with bacteria such as *S. aureus* or *Escherichia coli* or treated with lipopolysaccharide, their endogenous GSL synthesis rises, and they can stimulate CD1-restricted T cells that react to self-GSLs in the absence of exogenous lipid antigens^107^. Such new synthesis of endogenous lipids is driven by TLR triggering of APCs^179,180^: Paget et al. showed that when DCs are stimulated via TLR9, the expression of several sialyltransferases that participate in GSL synthesis is upregulated. The CD1-mediated presentation of charged β-linked GSLs by the DCs plus their production of type-I IFN then activates *i*NKT cells to secrete IFN-γ^180^. However, although treating DCs with GSL synthesis inhibitors reduced *i*NKT cell activation, it was not completely inhibited^179,180^, which suggests that other lipids also contribute to the repertoire of self-lipid antigens that promote *i*NKT cell activation in these settings.

Many microorganisms are able to evade host innate immune responses via a variety of mechanisms. Concerning host CD1-restricted T cell responses, these can be blocked by decreasing CD1 levels. Viruses can achieve this by reducing CD1 mRNA or protein levels. For example, human cytomegalovirus encodes an IL-10 homolog that mimics endogenous IL-10 and reduces the transcription of group 1 CD1 molecules by human DCs^181^. Similarly, when Epstein‒Barr virus infects B cells, it produces lymphoid enhancer binding factor-1 from its own genome, which binds to the CD1d promoter and rapidly downregulates CD1d expression^182^. This virus also produces BGLF5, a viral alkaline exonuclease, which degrades mRNA and thereby helps downregulate CD1d expression^183^. Moreover, human papillomavirus E5 interacts with CNX, which induces the translocation of CD1d from the ER to the cytosol; this induces CD1d proteasomal degradation and thereby downregulates CD1d levels^184^.

Viruses also block CD1-restricted T cell responses by interfering with CD1 recycling. In Kaposi sarcoma-associated herpesvirus, this is mediated by the two modulator-of-immune recognition (MIR) genes in the viral genome. When the MIR genes are expressed, the proteins ubiquitinate the lysine residues in the cytoplasmic tail of CD1d, which triggers CD1d internalization from the cell surface^185^. Similarly, herpes simplex virus-1 (HSV1) prevented recycled CD1d molecules from returning to the cell surface by trapping them in lysosome-like structures^186^. This may confer an adaptive advantage on this virus; when human DCs were infected in vitro with HSV, their surface group 1 and 2 CD1 levels were elevated when the HSV titers were low. However, when the titers were high, surface CD1 levels were reduced. This was linked to the disruption of the CD1-recycling machinery in DCs^187^.

## Concluding remarks

Here, we reviewed the CD1-mediated lipid antigen presentation system in the context of health and disease. To generate accurate CD1-restricted T cell responses, this antigen presentation system requires the finely tuned and dynamically interactive activities of numerous factors. If even one factor is deficient, mutated, or dysregulated by environmental insults or metabolic imbalances, beneficial CD1-mediated immune responses may be abrogated and/or pathogenic responses may emerge. These changes could promote, or even drive, the immunopathology of mucosal tissue diseases.

While CD1-restricted T cell responses contribute to skin, respiratory, and intestinal diseases and can be subverted by infectious organisms, the amount of research effort into these responses remains low at present. This reflects technical difficulties in isolating and identifying lipid antigens and the lack of group 1 CD1 in mice. Nonetheless, the development of lipidomics, 3D-culture systems, organoids, and CD1-tetramers has opened the field up, with the result that we are soon likely to find that CD1-mediated immune responses participate in many immunopathologies of mucosal tissues via hitherto unrecognized mechanisms.

Finally, our review showed that cellular metabolism can play key roles in the CD1 lipid-antigen presentation system. PPARγ and AMPK, which play very important roles in normal cellular metabolism, drive CD1 expression. Stressors that alter ER or mitochondrial function can alter the metabolism of APCs, thereby promoting the abnormal presentation of self-lipids or the production of unusual self-lipids. Moreover, our review emphasizes changes in the CD1-mediated antigen presentation system in mucosal diseases and microorganism infections (Table 2). These interactions between the CD1 antigen presentation system and cellular metabolism and the understanding of CD1-mediated immune responses in the pathogenesis of mucosal tissues could be targets of novel therapies that ameliorate or prevent numerous diseases.

**Table 2:** Influence of altered metabolism and diseases on the CD1-mediated antigen presentation system.

Condition		Species	APC type	Influence on CD1-mediated antigen presentation system	Changes of secreted cytokines by CD1-responding T cells	References
moDC differentiation		Human	moDCs	CD1d transcription ↑	•	^139,140^
				Group 1 CD1 transcription ↓		
Activated B cells	CD40L-mediated activation	Human	B cells	CD1c and CD1d transcription ↓		^144^
	Only BCR-mediated activation			CD1c expression ↑, CD1d expression ↓		
	BCR and CD40L simultaneous activation					
Cellular stress	ER stress	Human Mouse	Macrophage cell lines	Endosomal/lysosomal recycling of CD1d ↑	IL-2, IFN-γ↑	^104,106^
			DCs	Actin cytoskeletal remodeling		
			Macrophages	Neutral lipids loaded onto CD1d ↑		
	Mitochondrial stress	Human	CD1b^+^ cell lines	Phosphatidylglycerol synthesis ↑	•	^105^
Skin diseases	Bee and wasp venom allergy	Human	CD1a^+^ cell lines	Generation of neolipid antigens ↑	IFN-γ, GM-CSF, IL-13↑	^108,149^
			moDCs			
			LC-like cells			
	AD	Human	CD1a^+^ cell lines	Generation of neolipid antigens ↑	IFN-γ, GM-CSF, IL-13↑	^109,152^
			moDCs			
			LC-like cells			
	Psoriasis	Human	CD1a^+^ cell lines	Generation of neolipid antigens ↑	IFN-γ, IL-17A, lL-22↑	^153,155^
		Human	Dermal DCs	CD1b expression ↑	IL-17A↑	^157^
		Transgenic mouse				
		Human	KCs	CD1d expression ↑	IFN-γ, IL-13↑	^26,158^
			Dermal DCs			
	Allergic CD	Human	moDCs	CD1 synthesis ↑	IFN-γ, GM-CSF↑	^161^
			CD1a^+^ monocytic cell lines			
			CD1d^+^ monocytic cell lines			
	Lipophilic environmental pollutant-induced CD	Human	DCs	CD1a and CD1d trafficking ↓	IFN-γ↓	^162^
Pulmonary disease	Type 2 asthma	Human	DCs	CD1a and CD1c expression ↑	•	^167,168^
	COPD	Human	Alveolar macrophages	CD1b expression ↑	•	^23^
			Monocyte-derived macrophages			
		Mouse	Alveolar macrophages	CD1d expression ↑	IFN-γ, IL-17A↑	^170^
			Airway epithelial cells			
Gastrointestinal diseases	ABL	Human	DCs	Proteasomal degradation of group 1 CD1 ↑	IFN-γ↓	^171^
				Blocked antigens loading onto CD1d		
	Oxazolone-induced colitis	Mouse	IECs	Blocked CD1d-mediated antigen presentation	•	^121^
	Oxazolone-induced ulcerative colitis	Mouse	IECs	CD1d transcription ↑	IL-13 ↓ , IL-1β ↓ , IL-10 ↑	^174^
	Ulcerative colitis	Human	Macrophages	CD1a expression ↑	•	^22^
			Monocytes			
Bacterial infection	*Mycobacterium tuberculosis*	Human	Monocytes	Group 1 CD1 transcription and expression ↑	IL-2↑	^175^
				CD1d transcription and expression ↓	•	
		Mouse	Macrophages	CD1d expression ↑	IL-4↑	^178^
			CD1d^+^ cell lines			
	*Mycobacterium leprae*	Human	DCs	CD1b expression ↑	IFN-γ↓	^176,177^
				CD1b-mediated antigen presentation ↓		
	*Escherichia coli*	Human	DCs	Synthesis of GSL ↑	IFN-γ, IL-4↑	^107,179,180^
	*Bacillus subtilis*					
	*Staphylococcus aureus*					
	*Mycobacterium bovis-Bacillus Calmette Guerin*					
Viral infection	Lymphocytic choriomeningitis virus	Mouse	CD1d^+^ cell lines	CD1d-mediated antigen presentation ↑	IL-2, IFN-γ↑	^146^
			Thymocytes			
	HCMV	Human	DCs	Group 1 CD1 transcription and expression ↓	•	^181^
	EBV	Human	B cells	CD1d expression ↓	•	^182,183^
	Human papillomavirus E5	Human	CD1d^+^ cell lines	Translocated CD1d from ER to cytosol	•	^184^
	Kaposi sarcoma-associated herpesvirus	Human	CD1d^+^ B cell lines	CD1d internalization **↑**	IFN-γ↓	^185^
	HSV-1	Human	DCs	CD1d recycling ↓	IFN-γ↓	^186^
